# Conservation of parasites

**DOI:** 10.1016/j.ijppaw.2021.01.011

**Published:** 2021-02-09

**Authors:** Donald A. Windsor

**Affiliations:** Ronin Institute, PO Box 604, Norwich, NY, USA

**Keywords:** Parasites, Species

Conservation of parasites had a slow beginning, Windsor (1990, 1995) but is now a thriving trend, Dougherty et al. (2016). However, I worry that many biologists who are willing to conserve parasites may change their minds when they find out how nasty some parasites are.

Consequently, it is critical that conservationists realize that there are two levels at which parasites must be considered: the individual and the species.

Here is what must be emphasized. Species evolve. Individuals do not, Mayr (2001).

Parasites, as individuals, harm their hosts, by definition.
Parasites, as species, actually benefit their host species.

At the species level, parasites benefit their hosts by enabling them to survive. Parasites help manage the ecosystems in which the hosts live. When competition and predation do not control population sizes, parasites (including pathogenic microbes) take over, thus preventing monocultures. Without suitable ecosystems, the hosts would have to either go extinct or evolve into new species, Windsor (2016).

Without parasites, ecosystems would deteriorate into just a few, vast monocultures of very aggressively invasive species. Biodiversity is due to parasites.

A good everyday, non-biological example is taxes. Taxes harm individual taxpayers, but at a higher level, taxes benefit society.

We do not have to do much to conserve parasites. Just conserve their hosts and the parasites will be carried along. However, what we have to do is quit removing parasites when rehabilitated organisms are released back into the environment. And most importantly, we have to permit diseases to run their course; this will be very difficult and even impossible in some cases, such as zoonoses. Diseases are like wildfires, part of nature.

Every host species that is alive today, evolved with parasites. There are no host species currently alive that have no parasite species. Most of the species on Earth are parasites, Windsor (1998).
